# mfSBA: Multifractal analysis of spatial patterns in ecological communities

**DOI:** 10.12688/f1000research.3-14.v2

**Published:** 2014-04-07

**Authors:** Leonardo A. Saravia

**Affiliations:** 1 Instituto de Ciencias Básicas, Universidad Nacional de General Sarmiento, Buenos Aires, Argentina

## Abstract

Multifractals have been applied to characterize complex communities in a spatial context. They were developed for nonlinear systems and are particularly suited to capture multiplicative processes observed in ecological systems. Multifractals characterize variability in a scale-independent way within an experimental range. I have developed an open-source software package to estimate multifractals using a box-counting algorithm (available from https://github.com/lsaravia/mfsba and permanently available at doi: 10.5281/zenodo.8481). The software is specially designed for two dimensional (2D) images such as the ones obtained from remote sensing, but other 2D data types can also be analyzed. Additionally I developed a new metric to analyze

multispecies spatial patterns with multifractals: spatial rank surface, which is included in the software.

## Introduction

Multifractals and fractals are related techniques mainly used in physics to characterize the scaling behavior of a system; they differ in that fractals look at the geometry of presence/absence patterns, while multifractals look at the arrangement of quantities such as population densities or biomass ^1^. Scaling laws are an emergent general feature of ecological systems, and there is no *a priori* reason that power laws apply to ecological communities. If they do apply, they reflect constraints in their organization that can provide clues about the underlying mechanisms ^2, 3^. For example: in semi-arid vegetation patterns, power-laws are produced by facilitative interactions between plants against water scarcity ^4, 5^. In inter-tidal mudflat ecosystems the loss of power-law patterns is indicative of a degradation of the system ^6^. Changes in power-law vegetation patterns can signal the transition from a facilitation-dominated regime to a competition-dominated one ^7^. The previous examples deal with patch-size distributions but in several cases the definition of patches is not simple. Such cases are handled naturally by multifractals because they use densities or biomass directly.

Multifractals require that the object under study should be statistically self-similar, which means that a power-law could be fitted to data in a range of scales. But that does not mean that the power-law must be the best possible model. We can analyze the data without claiming that it is an exact multifractal ^8^. One of the advantages of multifractals is that they require fewer conditions on data than more classical statistics such as autocorrelation and variograms. These usually require isotropy and stationarity ^9^ but multifractals can be used with anisotropic data ^10^ and are inherently non-stationary ^11, 12^. Anisotropy and non-stationarity are often seen in spatial ecological distributions ^13^.

Multifractals are associated with systems governed by random multiplicative processes ^14^. In ecological systems, these processes can be given as the interaction of survival probabilities and compound growth ^15^. Moreover, the presence of multiplicative process is argued to produce the log-normal-like shape of species-abundance distributions ^16^. Also, random processes with spatial correlations can generate multifractals ^14^; these kind of processes are part of neutral community models ^17, 18^ and are observed in natural communities ^19^. Thus there are *a priori* reasons to think that multifractals can be applied to spatial ecological data. Indeed, they have been applied to vegetal communities ^20^, tropical forest ^21^, microphytobentos and periphyton biomass patterns ^1, 22^, and to the characterization of species-area relationships ^23– 25^.

Rank-abundance distributions are a representation of species-abundance distributions (SAD) that are a classical description of communities ^26^. These have been used to compare different communities and to compare models and data, but different mechanisms can produce nearly identical SADs ^27^. SADs are often presented using rank-abundance diagrams (RAD) where the log-abundance is plotted on the y-axis vs. rank on the x-axis ^26^. RADs are equivalent to cumulative distributions ^28^ and thus are a robust way to visualize the SAD without losing information ^29^. If the rank of each species is incorporated in its spatial distribution, it forms a surface: the species-rank surface (SRS). This SRS can be analyzed and compared using multifractals.

Here I present an open source software package that can be used for quantitative multifractal analysis (MFA) of densities, biomass or other continuous variables distributed in space. In addition the software can analyze SRS using MFA, though this kind of analysis is completely novel. A detailed description of the advantages of using SRS and MFA is outside the scope of this work and will be presented elsewhere. This software represents a step to make easier the use of multifractals for spatial pattern analysis in ecology. One of its advantages is that it can be integrated with another widely used software for quantitative analysis: the R statistical software ^30^.

## Multifractal analysis

Several good introductions to multifractal methods applied to ecology are available ^20, 31^; thus I will only give a brief overview. Multifractals analyze the scaling properties of quantities distributed in a space that we assume to be two dimensional (a plane), but MFA can be used with one dimensional (time series) or three dimensional data ^32^. A classical way to characterize multifractals is using the generalized dimensions *D _q_* ^33^, also called Renyi dimensions ^34^. *D _q_* has been used to portray the probabilistic structure of attractors derived from dynamical systems ^10^.

Another way to characterize multifractals is using the so called spectrum of singularities. This spectrum describes multifractals as interwoven sets each one with a singularity exponent *α* and a fractal dimension *f*( *α*) ^35^. The two multifractal representations are equivalent, they display the same information in a different format. But with the spectrum of singularities, two quantities are estimated ( *α* & *f* ( *α*)) from data and are obtained with error. Instead, with generalized dimension only one quantity is estimated *D _q_*, thus this method is preferred for statistical comparisons.

The multifractal spectra *D _q_*, is related to the Hill’s generalized diversity index *N _q_*, where *q* is an arbitrary real number ^36^. There are special cases of *q* that are of common use in ecology: for *q* = 0 *N _q_* corresponds to the number of species, *q* = 1 to the Shannon index, and *q* = 2 to the reciprocal of the Simpson’s diversity index. Thus *N _q_* defines different diversity measures as *q* changes, which vary in their sensitivity to rare species. In a similar way *D _q_* focuses on regions of the plane with higher densities if *q* is greater and in regions with lower densities if *q* is lower or negative.

There are two important differences, the first is that *N _q_* is calculated at one predetermined scale of measurement, and *D _q_* is related to how *N _q_* changes with scale ^37^. The second difference is that *D _q_* can be calculated on any quantity distributed in space, not only the number of species. The multifractal formalism was originally developed for additive quantities ^38^, and was later extended to non-additive quantities ^39^. This means that to apply MFA, the quantity distributed over space must increase in a mathematical sense. For example, if you have ten species in an area A1 and ten species in an area A2 the number of species in the sum A1+A2 will be greater than or equal to ten. If the number of species were additive the sum A1+A2 has to be 20, but that is not generally true. A limitation of mfSBA in its present version is that it only estimates multifractal spectra for additive quantities.

## Estimation

To estimate multifractal spectra I used the method of moments based on box-counting ^38^. I estimate generalized dimensions and the spectrum of singularities at the same time using the canonical method ^35^. Here I describe only the *D _q_* estimation; the steps for *α* and *f* ( *α*) estimation are identical (only the formulae to calculate the quantities are different and can be found in the appendix of Saravia *et al.* (2012) ^1^).

The spatial distribution that we are analyzing is covered with a grid, which is divided into *N* ( *∈*) squares of side *∈*. The contents of each square is called *μ _i_*( *∈*). Then the so called partition function is computed as:

$$Z_{q}(\epsilon )=\sum_{i}^{N(\epsilon )}{\left(\mu_{i}(\epsilon )\right)}^{q}\;\;\;\;\;(1)$$

Where *q* is called moment order. The operation is performed for different values of *∈* and *q*, within a predetermined range. The generalized dimension is calculated as:

$$D_{q}=\frac{1}{q-1}\underset{\epsilon \to 0}{\lim}\frac{\log(Z_{q}(\epsilon ))}{\log\epsilon }\;\;\;\;\;(2)$$

When *q* = 1, the denominator of the first term in *D _q_* is undefined, so it must be replaced by the following expression:

$$D_{q}=\underset{\epsilon \to 0}{\lim}\frac{\sum_{i}^{N(\epsilon )}\mu_{i}(\epsilon )\log(\mu_{i}(\epsilon ))}{\log\epsilon }\;\;\;\;\;(3)$$

**Figure 1.**
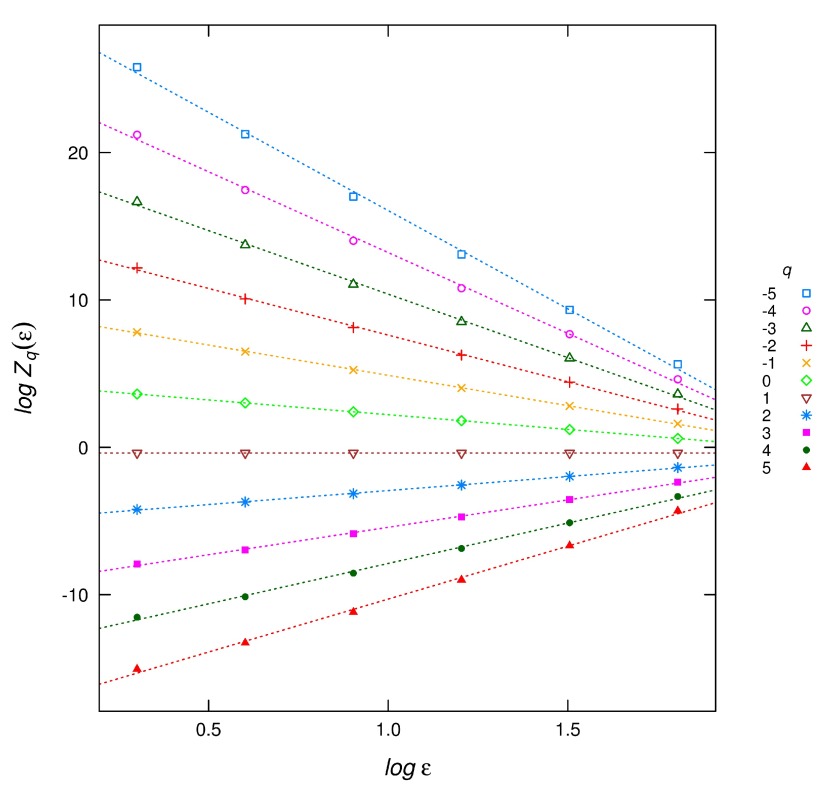
Plot of the linear regressions for different *q* used to estimate the *D _q_* multifractal spectrum.

**Figure 2.**
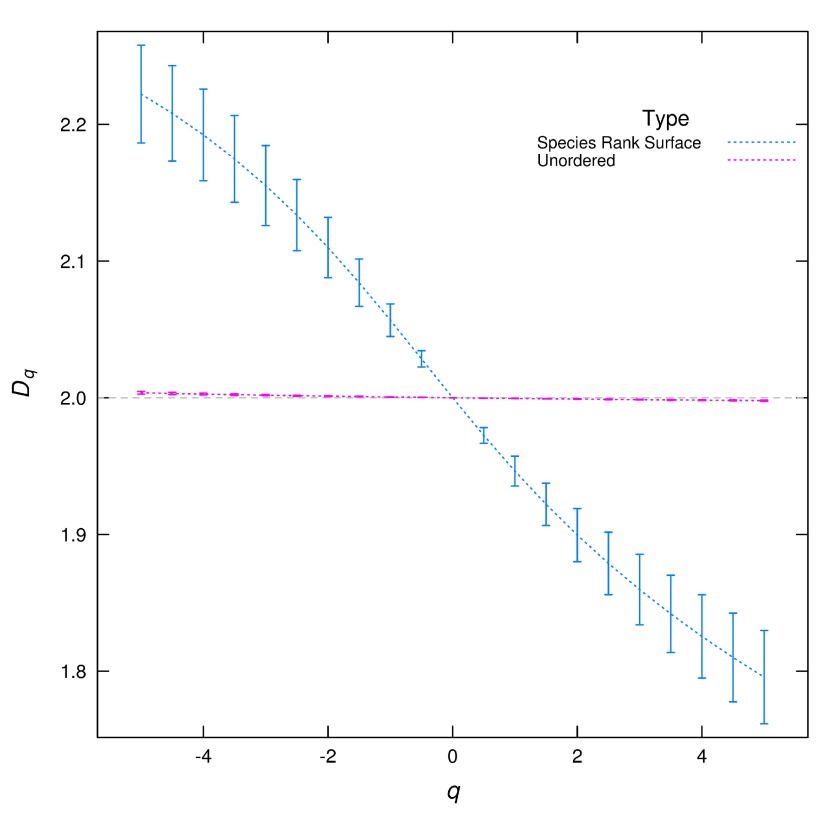
*D _q_* multifractal spectrum calculated from species spatial distributions. If the multispecies distribution is analyzed unordered (with species numbers assigned by the simulation software) the *D _q_* is almost flat corresponding to a uniform plus random noise distribution. But when the species rank surface (SRS) is used the *D _q_* spectrum has a wide range of values, corresponding with a highly heterogeneous distribution, formed of valleys for the most abundant species and peaks for rare species. The error bars are the standard deviation obtained from the linear regressions used to estimate *D _q_*.

In practical cases, as the limit can not be assessed, the dimensions are estimated as the slope of *log*( *Z _q_*) versus *log*( *∈*) ( Figure 1) divided by *q* – 1 as shown in equation (1). This is done for different *q*, provided that it is a real number which yields a graph of *D _q_* in terms of *q*, called the spectrum of generalized dimensions ( Figure 2).

To be an approximate multifractal, the relationship *log*( *Z _q_*) versus *log*( *∈*) should be well described by a linear relationship, although a linear relationship with superimposed oscillations is also acceptable ^31^. A range of *q* and *∈* is fixed and then *D _q_* is estimated using linear regressions. The coefficient of determination ( *R* ^2^) can be used as a descriptive measure of goodness of fit ^23^.

## Use of mfSBA software

The software was built and tested under Ubuntu 12.04 LTS Linux environment, using the GNU C++ compiler (v4.6.3). It requires the libtiff library for reading tiff images ( http://www.libtiff.org/). It can be compiled under Windows environments using the GNU compiler and utilities for that operative system ( http://www.mingw.org/).

You can download or clone mfSBA from https://github.com/lsaravia/mfsba (using **git clone** or as a zip file) and build it using the make utility.

**make -f mfSBA.mak**

You can run it from the command line using the following command structure:

**mfSBA inputFile qFile minBox maxBox numBoxSizes option**

the parameters are:

- **inputFile:** this file can have only two formats: 1) one-layer tiff 2) “sed” file format. Sed is an ascii format I invented to use with my own stochastic cellular automata models to represent a square grid of values. It has a header of two lines: the first line describes the two dimensions X Y of the data, and the second line describes the type of data. For this program, the type must be BI, which means that the values stored in the grid are real numbers with double precision. See the example file with a “.sed” extension.
- **qFile:** this is a sed file with a vector of values representing the q’s used to calculate the multifractal spectrum.
- **minBox,maxBox,numBoxSizes:** Minimum box size, maximum box size and maximum number of box sizes. The program uses box sizes in powers of two: if **maxBox** is greater than half of the image size, it is set to that value. If the number of boxes between **minBox** and **maxBox** is greater than **numBoxSizes**, the latter number of boxes will be used, discarding the biggest ones.
- **option:** is an upper case character with four possibilities: N,S,D,A.
      – N: analyze the input file as is.
      – S: normalize the input file then analyze it. Normalization is done summing all the pixels values and dividing each pixel by that total. After that the sum of all values is one.
      – D: add 1 to all the pixels then normalize as in S.
      – A: normalize as in S and save the normalized image as a sed file.

Examples of input files are included with the source code, thus after compiling you could run the following command assuming a linux system:

**./mfSBA b4-991008bio.sed q21.sed 2 256 20 S**

## Output

The program generates four output files, attaching a prefix to the original input file name:

- *t.inputFile*: this file has a header line with field names and *q* + 2 columns. The first two columns are the box sizes and log box sizes used in the estimation. After that, each column corresponds to log ( *Z _q_* ( *∈*)) of equation (2) with the *q*’s specified in the **qFile**. This file could be used to visually check the linearity assumptions to calculate *D _q_* as in Figure 1.
- *a.inputFile* & *f.inputFile*: are similar to the previous file, but used to calculate *α* and *f* ( *α*). The formulae are described in the appendix of Saravia *et al.* (2012) ^1^.
- *s.inputFile*: this file has a header line with field names and 10 columns. The first column is *q*. The second column is called Tau and is the result of the regression to calculate the limit in equation 2. Thus to obtain *D _q_* we have to divide it by *q −* 1, except in the case of *q* = 1 that we take the value of the next column to get *D _q_*. The third column is the value of *α* and the forth column *f* ( *α*). After that, columns are the corresponding coefficients of determination *R* ^2^ and standard deviations.

## Species rank surface

I propose to extend the analysis of SAD attaching the rank of each species to its spatial distribution. In this way, the multivariate spatial distribution of all species can be summarized into a univariate distribution. I called this spatial distribution the species-rank surface (SRS), and it can be analyzed and compared using MFA. To construct the SRS, I first calculate the rank-ordering of the species by their abundance from biggest to smallest, starting from one. Then the rank is assigned to the spatial position of the individuals of each species, forming a surface. This landscape has valleys formed by the most abundant species and peaks determined by the rarest species, and the standard MFA can be applied. SRS is additive, in the sense explained earlier, because ranks are not recalculated when the scale changes. The program used to calculate this is called multiSpeciesSBA, and is included with the mfSBA source code. You can compile it using the following command:

**make -f multiSpeciesSBA.mak**

Then all the input files and parameters are identical to mfSBA except that the program expects an **inputFile** containing a multispecies distribution. So the **inputFile** should be composed of integer numbers each one representing one species. An example of a sed file with a multispecies spatial distribution is given in t64-0100.sed, this file was obtained using a spatially explicit neutral model with 64 species (available at https://github.com/lsaravia/neutral). You can use the following command to perform the MFA:

**./multiSpeciesSBA t64-0100.sed q21.sed 2 128 20 N**

## A confidence envelope for spatial randomness

To determine if *D _q_* spectra are different from ones produced by a random spatial distribution of the quantity analyzed, I developed a randomization test. I shuffled all the positions of the original distribution and recalculated *D _q_*. The procedure is repeated many times (e.g. 1000) and the highest and lowest tails determine a confidence envelope ^40^. If the actual values of *D _q_* falls outside the envelope the spatial pattern is not random for that particular *q*. This program can be compiled using the following command:

**make -f mfSBArnz.mak**

The command to run the analysis has a similar structure to the previous:

**mfSBArnz inputFile outFile qFile minBox maxBox numBoxSizes option numSimul P**

All the parameters previously mentioned have the same meaning, I will explain only the new ones:

- **outFile:** this is the name of a file with all the *D _q_* calculations specified with the numSimul parameter. It has the same format as the s.inputFile with an additional first column labeling the original *D _q_* or a randomization. This file could be used to calculate the confidence envelope with a different P without doing the randomizations again. The program generates another file with an added “.rnz” extension, which contains the summarized results: the first row contains the number of randomizations and the size of the tail, the second row the p-value requested, the third row the field names, and from the fourth row there are five columns: the original values of *q* and *D _q_*, two colums representing the randomization envelope at the requested p-level (DqMax & DqMin) and a column with 1/-1 indicating if the original *D _q_* falls outside the envelope or 0 if it was inside the envelope.
- **numSimul:** the number of randomizations, a bigger number will give a more accurate confidence envelope at the requested p-level ^41^.
- **P:** a two tailed p-value for the confidence envelope, that is the chance than *D _q_* falls outside the envelope if the spatial pattern is random (Type I error).

An example of this analysis can be performed with the following command:

**./mfSBArnz t64-0100.sed t64-0100 q21.sed 2 256 20 S 1000 0.05**

If the software is used under Windows the name of the executable program should be changed, deleting “./” and adding the “.exe” extension, for this last example it should be mfSBArnz.exe.

## R integration

Included with the source is a set of functions as an example to integrate the mfSBA software with the R language ( http://www.r-project.org/). You should have compiled or downloaded the executables in the same folder as the R scripts. Then you can load the functions inside R with:

**source('Fun_MFA.r')**

and then run the same given examples:

**dq1<− calcDq_mfSBA("b4-991008bio.sed","q21.sed 2 256 20 S")**

An interesting example is to compare the *D _q_* from the example multispecies spatial distribution untransformed

**dq1<− calcDq_mfSBA("t64-0100.sed","q21.sed 2 512 20 S",T)**

**dq1$Site <− "Untransformed"**

with the *D _q_* from SRS

**dq<− calcDq_multiSBA("t64-0100.sed","q21.sed 2 512 20 S",T)**

**dq$Site <− "Species Rank Surface"**

**dq <− rbind(dq,dq1)**

and plot *D _q_* with

**plot_DqCI(dq)**

**Supplementary Figure 1.**
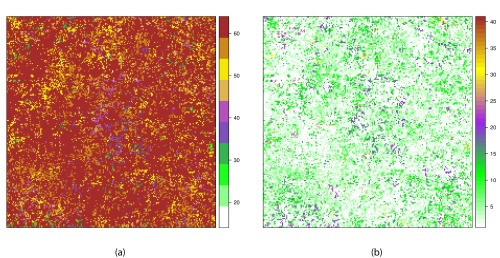
Comparison of a multiespecies spatial distribution generated by a neutral model with 64 possible species. Both figures represent the same species distribution but with diferent numeric values assigned to species. ( **a**) The values are assigned by the simulation program. The most abundant species have higher values and the range 1–64 represents all posible species. ( **b**) Spatial Rank Surface: each species is assigned to its rank as explained in the main text: the most abundant species has the lowest value and represent valleys, rare species represent peaks. The range is lower because some species did not appear.

In this plot ( Figure 2), we can see that the two *D _q_* spectra are different. The *D _q_* calculated from the unordered distribution is nearly flat, this corresponds to an almost constant spatial distribution with uncorrelated random noise. The structure of SRS is lost when species are assigned with a different order, valleys are formed by the most abundant species and peaks of rare species are destroyed. This is similar to comparing a RAD with a figure made with the ranks disordered on the x axis. Although the species distribution is the same, the two surfaces are different ( Supplementary Figure 1), which is reflected in their *D _q_* spectra.

The plot of the *t.inputFile* ( Figure 1) gives a visual check of the regressions to obtain *D _q_*:

**plotDqFit("t.t64-0100.sed","q21.sed")**

additionally the *R* ^2^ values could be easily checked:

**hist(dq1$R.Dq)**

All the examples and more graphics are included in the file testMFA.r, you should change this file to reflect the folder where you downloaded the software.

## Conclusion

The multifractal spectrum can be used to describe and compare spatial patterns of biomass, density, height, point patterns, or any continuous variable. The condition is that the distribution of the variable in space must be additive. The mfSBA software is especially useful for remote sensing data because it can be used with tiff images. Multifractal patterns could be produced by the existence of multiplicative interaction between species and by spatially correlated random processes such as dispersal and growth ^14^. Plant and animal species are generally aggregated in space thus is very likely that multifractal analysis can be used in a wide range of cases.

The analysis of SRS using *D _q_* adds a new dimension to the comparison of species spatial distributions, because it can be used to compare spatial distributions of all species at the same time and also the abundances are accounted. An exploration of the results of different spatial patterns should be needed as a continuation of the present work.

The analysis of SRS using *D _q_* adds a new dimension to the comparison of species spatial distributions, because it can be used to compare spatial distributions of all species at the same time and as the species ranks are used, this is a spatial version of SAD. The method presented here analyzes SRS as a surface, which is different to SAR and to multifractals calculated with the number of species as in Borda-de-Agua (2002) ^23^. An exploration of SRS analysis resulting from different spatial patterns combined with different SADs will be needed as a continuation of the present work. Also the software should be extended to calculate MFA for non-additive distributions such as the number of species.

The software presented here is oriented to obtain multifractal spectra for comparisons, rather than to obtain the true value. While the estimation methods used in mfSBA could be improved ^31, 42^, it has been used without trouble with the kind of data obtained in ecological studies ^1, 43^.

Multifractals can be successfully used to analyze several aspects of community spatial structure. With the advent of the big data era in ecology ^44^ and the use of new technology to acquire spatial data ^45^, new methods to analyze complex data sets are needed and multifractals could be an interesting addition to the ecologist’s toolbox.

## Software availability

Zenodo: Multifractal estimation using a standard box-counting algorithm: version 2, doi: 10.5281/zenodo.8481 ^46^

GitHub: Multifractal estimation using a standard box counting algorithm, https://github.com/lsaravia/mfsba
