# Supplementary material for: mfSBA: Multifractal analysis of spatial patterns in ecological communities
Source: F1000Res. 2014 Apr 7;3:14. Originally published 2014 Jan 16. [Version 2] doi: 10.12688/f1000research.3-14.v2 (PMC4197745; doi:10.12688/f1000research.3-14.v2)
Supplement: Peer review history

# Peer review: Referee response for version 2

**DOI:** 10.5256/f1000research.4039.r4385

**Published:** 2014-06-26

**Authors:** Edmund Hart

**Affiliations:** 1 National Ecological Observatory Network, Boulder, CO, USA

The author has addressed a majority of my concerns.  I felt the explanations and cases used were much clearer with this set of revisions than in the past, and I can foresee how I could use the software.  I also believe that providing windows binaries will also significantly help users.  My only trouble spot was building the package from source and it's dependency on libtiff.  However I think that is an issue that can be resolved on the issues page of the repo.  My one thought for what might improve the paper for the more novice users would be to include that issues could be posted on the github repo, not just cloning the source.  Other than that I think the paper is substantially improved and is strong work.

I have read this submission. I believe that I have an appropriate level of expertise to confirm that it is of an acceptable scientific standard.

# Peer review: Referee response for version 2

**DOI:** 10.5256/f1000research.4039.r4386

**Published:** 2014-05-22

**Authors:** Yuxin Zhang

**Affiliations:** 1 State Key Laboratory of Urban and Regional Ecology, Chinese Academy of Sciences, Bejing, China

I tested the revised R script, it works well under Window OS.

I have read this submission. I believe that I have an appropriate level of expertise to confirm that it is of an acceptable scientific standard.

# Peer review: Referee response for version 1

**DOI:** 10.5256/f1000research.3496.r3330

**Published:** 2014-02-14

**Authors:** Edmund Hart

**Affiliations:** 1 National Ecological Observatory Network, Boulder, CO, USA

I think this software package will make a needed contribution to ecology.  It meets are criterion for technical merit.  However I believe the paper can still be substantially improved.  There are two aspects which will benefit from revision.

**Main text**

*Introduction:*

I think the introduction would benefit greatly from clear examples of the benefits and uses of mfSBA.  It seems that the author should address both the benefits of multifractal analysis as well as where the SRS improves upon comparisons made by SAD's. It seems that the author has already made some publications that could address this, and it would be useful to have a brief explanation.  

In general I found the Introduction lacking specificity. For instance the author states in the first paragraph: "If they do apply, they reflect constraints in their organization that can provide clues about the underlying mechanism"  What constraints?  What underlying mechanism is the author referring to?  How  does it provide clues about this these mechanisms?

I finished reading the introduction and was unclear both about what multifractal were, and why I would want to use them.

*Methods and conclusion*

I think the most important part here is a clear explanation of the quantities of interest that I can get from mfSBA, and how to interpret them. While parameters like slope and intercept seem obvious to most people now, I had no idea how to interpret q or D_q, or how I would use them.  

Furthermore the author focuses on the ability of this new method to make comparisons, stating: "The analysis of SRS using *Dq* adds a new dimension to the comparison of species spatial distributions, because it can be used to compare spatial distributions of all species at the same time and also the abundances are accounted."

However, the comparison the author makes is very unclear.  It would be nice to see a comparison where simulation conditions were clearly made, and perhaps even performed in R so users could tweak them.  Figure 2 provides output for a comparison, but it's unclear how to interpret that comparison.  In summary the main text would benefit from a clearer presentation of use cases, what parameters are being estimated, and how to understand those parameters in different scenarios, both simulated and real data.

**Software packaging.**

I think the average ecologist will find this package still rather intimidating.  It requires the installation of secondary libraries as well as a C compiler.  I wouldn't even know how to begin to install this on a windows machine.  I would suggest the author create an R package with precompiled binary libraries for windows and mac. This combined with an improved manuscript will provide ecologists with a powerful tool.

I have read this submission. I believe that I have an appropriate level of expertise to confirm that it is of an acceptable scientific standard, however I have significant reservations, as outlined above.

# Peer review: Referee response for version 1

**DOI:** 10.5256/f1000research.3496.r3170

**Published:** 2014-01-24

**Authors:** Yuxin Zhang

**Affiliations:** 1 State Key Laboratory of Urban and Regional Ecology, Chinese Academy of Sciences, Bejing, China

This is a useful Web Tool for the researcher, particular the ecologist in the community ecology, who wants to use the multifractal method describing the SAD and point pattern of species distribution. The paper is well written. However, the script (followed with the instruction in the “R integration” section) don't work well in Windows with R 3.0.2 (64bit). The author needs to revise the R scripts or provide detailed instruction on it, before the work is acceptable. Reports on the performance of the software in GNU for the Windows environment are also needed.

There are two minor comments:

1. The q is usually an integer in community ecology, because this will directly correspond to the Hill numbers. 
2. The range of q in this paper is from -5 to 5. Can the users of this tool set the range of q for their own preference? 

I have read this submission. I believe that I have an appropriate level of expertise to confirm that it is of an acceptable scientific standard, however I have significant reservations, as outlined above.
